# Kraepelin, the unitary psychosis theory and the classification in psychiatry

**DOI:** 10.1192/j.eurpsy.2023.1580

**Published:** 2023-07-19

**Authors:** J. J. Martínez Jambrina

**Affiliations:** PSYCHIATRY, HOSPITAL SAN AGUSTIN, AVILÉS, ASTURIAS, Spain

## Abstract

**Introduction:**

The history of psychiatry is the history of the unitary psychosis concept. Recent studies on this subject by prominent authors (Huda, Stanghellini, Broome, etc.) confirm that the problem has not been resolved but that it reappears now with more force from skeptical positions, based above all on the insufficiency of the classification criteria official (ICD-10, DSM-V). This skepticism has led to an attempt to make the line that separates normality from mental illness disappear with the weak argument that isolated psychotic symptoms are detected in the general population.The proposals to stop these movements that border on the denial of mental illness try to provide more information on the contextual and subjective factors of mental illness, thus reinforcing the so-called biopsychosocial model formulated by Engel in 1976, whose conceptual imprecision is largely responsible for the problems before indicated as well as other more serious ones for the organization of psychiatric care.

**Objectives:**

Point out the insufficiencies of the biopsychosocial model.-Point out the advantages of acquiring a classic evolutionary approach such as the one designed by Iván Pavlov besides the Volga and improved by some followers.-highlight the differences between researching in psychopathology, a true science, or doing it in clinical psychiatry, its practical application. This distinction is essential.

**Methods:**

The works of some authors who have approached this conflict with dedication and rigor will be reviewed.Research lines followed during last hundred years in psychiatry will be contrasted with the results obtained.

**Results:**

New points of view and new tools need to be incorporated to solve this conflict that confuses experts so much are proposed.Ways of working are indicated that should avoid confusion between psychopathology and clinical psychiatry

**Conclusions:**

A psychiatric diagnosis must be established on solid conceptual basis that we currently lack.-Both Kraepelin and Kurt Schneider are two key figures to recover and keep current in our daily practice.-The importance of patient’s subjectivity when taking an anamnesis of their problems seems very important. The question is how to manage that subjectivity in order to analyze it from a classical scientific model, Pavlov’s great desire.-A revisiting of Husserlian phenomenology is essential in the training plans of young psychiatrists and in daily psychiatric care. But this is not enough. We need new tools and new conceptual frameworks so that the phenomenological perspective can contribute to put light in problems as important as those generated by the constant change of diagnosis that is carried out with many patients.If we want a scientific psychiatry we cannot handle with tools that have failed since their creation.

**Disclosure of Interest:**

None Declared

